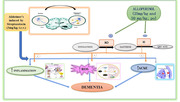# Exploring potential of Allopurinol in Dementia of Alzheimer’s type

**DOI:** 10.1002/alz.087850

**Published:** 2025-01-09

**Authors:** Amit Kumar, Diksha Sharma, Pragati Silakari, Amarjot Kaur Grewal

**Affiliations:** ^1^ Chitkara College of Pharmacy, Chitkara University, Rajpura, Punjab India

## Abstract

**Background:**

Dementia is a mental condition defined by a severe loss of intellectual ability that interferes with one’s occupational or social activities. The rapid increase in the number of patients with dementia and Alzheimer’s disease (AD) will result in tremendous consequences for our society and economy. Hypoxanthine is a purine compound that is implicated in the progression of AD. The conversion of hypoxanthine into uric acid by xanthine oxidase reductase (XOR) leads to the formation of superoxide species that increase oxidative stress. The aim of the present study is to investigate the protective effect of allopurinol on STZ‐induced Alzheimer’s‐type dementia.

**Method:**

A docking study was done to evaluate the interaction of allopurinol with AChE. Once the interaction was confirmed, the study used Swiss albino mice. The test drug was administered to STZ mice for a total of 7 days (16‐22 days). Animals were subjected to the Morris Water Maze (MWM) training trials for 4 days (Days 19‐22). On the 21^st^ and 22^nd^ days, we performed the step‐down passive avoidance test. On the 23^rd^ day, retrieval trials were conducted on MWM and step‐down passive avoidance, and animals were sacrificed and the whole brain extracted for biochemical and histological investigations.

**Result:**

STZ‐induced mice showed decreased performance in MWM, stepdown‐passive avoidance, and altered biochemical and inflammatory cytokines. The histopathological study revealed severe infiltration of neutrophils and amyloid deposition. In the study, allopurinol treatment mitigated STZ‐induced cognitive decline via behavioural, biochemical, and histopathological changes.

**Conclusion:**

The results suggest that the neuroprotective effects of allopurinol in STZ‐induced Alzheimer’s‐type dementia might be attributed to anticholinesterase, antioxidative, and anti‐inflammatory effects.